# Histologic Evaluation of Normobaric Oxygen Therapy Safety in an Animal Model

**Published:** 2012-08-25

**Authors:** Esmaeil Idani, Nastaran Ranjbari, Farideh Sharifipour, Ali Asghar Hemmati, Mohammad Malekahmadi

**Affiliations:** 1 Department of Pulmonology, Ahvaz Jundishapur University of Medical Sciences, Ahvaz, IR Iran; 2 Department of Pathology, Ahvaz Jundishapur University of Medical Sciences, Ahvaz, IR Iran; 3 Department of Ophthalmology, Ahvaz Jundishapur University of Medical Sciences, Ahvaz, IR Iran; 4 School of Pharmacy, Ahvaz Jundishapour University of Medical Sciences, Ahvaz, IR Iran

**Keywords:** Safety, Oxygen Inhalation Therapy, Rabbits

## Abstract

**Background:**

Oxygen therapy, as a therapeutic modality, can be used for long periods of times. However, it may be accompanied by potential complications and side effects.

**Objectives:**

To evaluate the side effects of normobaric oxygen therapy in rabbits.

**Materials and Methods:**

In a double-blind experiment, 28 white New Zealand rabbits were randomized into an oxygen treatment group (n = 14) and a control group (n = 14). The oxygen treatment group received 100% oxygen at a flow rate of 5 L/min for 1 h daily, for 1 month. The animals were euthanized at the end of the study, and following autopsy a histological evaluation was carried out to detect levels of oxygen toxicity in their; lungs, liver, brain, heart, kidney, eyes and spleen.

**Results:**

Histological evaluation revealed no evidence of toxicity in the examined tissues, compared with the control group.

**Conclusions:**

Oxygen therapy at a flow rate of 5 L/min for 1 h daily for 1 month had no systemic toxicity and it appears to be safe in rabbits.

## 1. Background

Oxygen therapy is used extensively as both an inpatient and outpatient therapeutic modality. Like any drug, oxygen has side effects and potential complications when given in excessive amounts (1). The age of the patient, normobaric versus hyperbaric oxygen therapy, method of oxygen delivery, flow rate, frequency and duration of treatment, may all play a role in the development of complications. Retinopathy of prematurity, bronchopulmonary dysplasia and possible transmission of infection to the lungs via the tubing and breathing apparatus, are potential side effects of treating premature infants with oxygen. According to human and animal studies, after inspiration of high concentrations of oxygen, a spectrum of lung injuries ranging from mild tracheobronchitis to diffuse alveolar damage can occur (2, 3, 4, 5, 6).

Oxidative damage may develop in any cell in the body, but the effects on the three most susceptible organs; central nervous system (CNS), lungs and eyes, are of primary concern (7, 8). It may also be implicated in; red blood cell destruction (hemolysis) (9), and damage to the liver (10), heart (11), endocrine glands (adrenal, gonads, and thyroid) (12, 13), or kidneys (14), and general damage to cells (15).

There is no single threshold of fraction of inspiratory oxygen (FIO2) defining a safe upper limit for the prevention of oxygen toxicity. The relative importance of the duration and magnitude of hyperoxic exposure has also not been clearly defined (16).

## 2. Objectives

Considering the expanding indications for oxygen therapy and concerns about its safety, we conducted an animal study to evaluate histological changes in the; lungs, liver, brain, heart, kidney, eyes, and spleen, following oxygen therapy in rabbits.

## 3. Materials and Methods

This study was performed at the Center for Animal Research, School of Pharmacy, Ahvaz University of Medical Sciences. The study protocol was in accordance with the guidelines for the care and use of laboratory animals. Twenty-eight healthy New Zealand white rabbits were used in this trial. The rabbits were fed with a standard caloric diet for their age. In order to find the best method for administering oxygen, we performed a pilot study in 2 groups, each consisting of 3 rabbits. For the first group, the rabbits were kept in restrainers, and oxygen was administered through a face mask. In the second group, we put each rabbit in a chamber with 2 holes, 1 for the entrance of oxygen and the other for the air to exit, to keep the condition normobaric. Holding the rabbits tightly in restrainers for 1 hour and using a simple mask was not a comfortable method for them, and they tried to escape. In the chamber, they remained calm and had no space to move freely. Therefore, we used this method.

In a double blind study, the rabbits were randomized using a randomization table into two groups; an oxygen treatment group (n = 14) and a control group (n = 14). The oxygen treatment group received 100% oxygen at a flow rate of 5 L/min for 1 h daily. Each rabbit was put into a small chamber as previously mentioned. The temperature in the chamber was maintained between 22–24° C, with a relative humidity between 60–70%. Arterial blood gas analysis was performed once for each rabbit, on arterial blood taken from the central auricular artery, before and immediately after, oxygen therapy. All experimental animals were continuously visually monitored throughout all exposures, for evidence of CNS toxicity. Possible signs of toxicity include; restlessness, rigidity, excessive cleaning movements, salivation, shaking, deep breathing movements, and convulsions. The observer was masked to the intervention. The rabbits were monitored for 30 days and then euthanized with an overdose of pentobarbital. Chest and peritoneal cavities were opened carefully. The brain, heart, lungs, spleen, kidney, liver and eyes were excised and after fixation in formalin 10%, a histologic evaluation was done by an expert pathologist (NR), who was also masked to the intervention.

## 4. Results

No abnormal behavior such as; restlessness, rigidity, salivation, and convulsions, was observed in either group over the study period. All rabbits behaved normally and tolerated the oxygen therapy well. No abnormal signs were detected. Arterial blood gas analysis performed on the blood taken from the central auricular artery showed an increase from a baseline of 79 ± 10 mm Hg before oxygen therapy to 180 ± 15 after oxygen therapy. After autopsy and histologic evaluation of their; lungs, heart, brain, kidney, spleen, liver and eyes, no abnormal changes were found and all of these tissues retained their normal architecture. Special stains, i.e., reticulin and trichrome were used and there were no parenchymal changes, necrosis, inflammation or structural abnormality, found in either of the groups.

## 5. Discussion

Oxygen is the first prerequisite of life, without which we cannot survive. The balance between oxygenic photosynthesis and aerobic respiration maintains homeostasis within our planet biosphere. In addition, it has therapeutic roles and is considered to be a drug when administered at higher concentrations than are usually present in nature (FIO2=21%). Oxygen therapy is, however, like a two-edged sword. On the one hand oxygen is essential for human survival and serves as a drug in certain conditions, while on the other hand it may become toxic at an elevated partial pressure. This can be hazardous, especially in intensive care units, where oxygen may be administered for days or even months.

Hyperoxia is poorly defined, but probably exists whenever oxygen tension exceeds 21% of inspiratory air. It appears to produce cellular injury through the increased production of reactive oxygen species (ROS) such as; the superoxide anion, hydroxyl radical, and hydrogen peroxide (17). ROS is a collective term for both oxygen radicals and certain oxidizing agents. ROS play a dual role in biological systems, having either harmful or beneficial effects on living systems (18). Oxygen free radicals may also promote a deleterious inflammatory response, leading to secondary tissue damage and/or apoptosis (19). Oxygen toxicity usually manifests in one of several forms including; CNS, pulmonary, and ocular manifestations (20).

Comroe et al. showed that many healthy individuals experience; substernal heaviness, pleuritic chest pain, cough, and dyspnea within 24 hours of breathing pure oxygen, and that these symptoms are probably due to a combination of tracheobronchitis and absorptive atelectasis (21). Sackner et al. reported that erythema and edema of the large airways can be observed bronchoscopically in the majority of patients treated with a FiO2 of 90% for six hours and this is thought to reflect hyperoxic bronchitis (22). In a study by Freeman et al. normobaric oxygen therapy with 85% oxygen continuously for 7 days, resulted in lung toxicity in rats (17). Demchenko et al. reported that following oxygen therapy for 56 hours, the lungs in some rats became grossly edematous and congested, and diffuse pulmonary hemorrhage was observed (23). In another study by Schaffner et al., after 2 weeks of oxygen therapy with a pressure of 258 mmHg, microscopically hepatic toxicity occurred in rabbits (10). Busing et al. showed that heart muscle necrosis, following normobaric hyperoxia as examined under a light microscope, occurred after 40 hours exposure in rabbits and this increased in intensity with a prolongation of the exposition time (24). Our study showed that intermittent systemic oxygen therapy at a flow of 5 L/min for 1 h daily for 1 month had no systemic toxicity on; lungs, brain, liver, spleen, kidney, heart or eyes in rabbits.
